# Extreme variation in migration strategies between and within wandering albatross populations during their sabbatical year, and their fitness consequences

**DOI:** 10.1038/srep08853

**Published:** 2015-03-09

**Authors:** Henri Weimerskirch, Karine Delord, Audrey Guitteaud, Richard A. Phillips, Patrick Pinet

**Affiliations:** 1 Centre d'Etudes Biologiques de Chizé, CNRS, 79360 Villiers en Bois, France; 2 British Antarctic Survey, Natural Environment Research Council, High Cross, Madingley Road, Cambridge CB3 0ET, UK

## Abstract

Migratory behavior, routes and zones used during the non-breeding season are assumed to have been selected to maximize fitness, and can lead to genetic differentiation. Yet, here we show that migration strategies differ markedly between and within two genetically similar populations of wandering albatross *Diomedea exulans* from the Crozet and Kerguelen archipelagos in the Indian Ocean. Wandering albatrosses usually breed biennially if successful, and during the sabbatical year, all birds from Kerguelen migrate to the Pacific Ocean, whereas most from Crozet are sedentary. Instead of taking the shortest routes, which would involve a return against headwinds, migratory birds fly with the westerly winds, requiring detours of 10,000 s km. In total, migrants circumnavigate Antarctica 2 to 3 times, covering more than 120,000 km in a single sabbatical year. Our results indicate strong links between migratory behavior and fitness; all birds from Kerguelen breed biennially, whereas a significant proportion of those from Crozet, especially females, are sedentary and breed in consecutive calendar years. To breed annually, these females temporarily change mate, but return to their original partner in the following year. This extreme variation in migratory behavior has important consequences in term of life history evolution and susceptibility to climate change and fisheries.

The annual cycles of species, populations and individuals can differ markedly, and whether animals are resident or migratory has major consequences for interactions and processes in local versus distant environments^1^, and, ultimately, on fitness^2^. Migration is usually considered to be a strategic response to spatially seasonal variation in the environment, allowing individuals to escape unfavorable conditions^3^. However, some populations show partial migration; some individuals migrate between habitats whereas others remain near the breeding colony, year-round^4^. The mechanisms that ultimately drive the decision to migrate or not remain controversial, and little empirical work has considered the potential consequences of partial migration^5^. In particular, the behavior of migrants is much harder to study than that of resident species, yet events during the non-breeding (migration) period are critical for population regulation^6^. In addition, migratory strategies are assumed to be optimal, minimizing travel duration and total energy expenditure^7^,^8^ so that survival and future reproductive performance are maximized^9^. The shortest route is not necessarily the best for terrestrial migrants, since environmental conditions en route, such as unfavourable winds, feeding opportunities or topographic obstacles may increase the overall costs of migration compared with an alternative, longer distance journey^7^.

This rationale also applies to the marine environment, where seabirds are known to undertake amongst the longest-distance migrations on earth^10^,^11^,^12^. Albatrosses and petrels are large, wide-ranging predators relying extensively on wind to conduct large scale movements during breeding, and to maximise flight efficiency^13^,^14^. Outside the breeding season they have potential for extensive dispersal^10^,^15^, but it remains unclear to what extent they optimise migratory routes. It is likely, for example, that they tailor their migration routes to avoid or utilise particular wind regimes^16^.

The wandering albatross (*Diomedea exulans*) is one of the most oceanic of all flying seabirds and has a distribution that includes vast areas of the Southern Ocean. Because its breeding season lasts around a year, birds usually take a sabbatical year between reproductive attempts^17^, although in some populations, a small proportion of individuals that have reared a chick will breed in the following year^18^. The few tracking studies published to date support earlier analyses of band recoveries, and indicate that during the intervening sabbatical years, birds move variable distances from the foraging grounds around the breeding colony to a range of nonbreeding destinations^19^,^20^,^21^. Nevertheless, the details of this migration, including the variation among individuals and populations, remains poorly documented^22^. Because of the reliance of albatrosses on wind for efficient flight, it is likely that migratory routes are influenced by wind conditions, and especially the strong circumpolar westerly flow^13^,^23^. Although they spend the long pre-breeding period foraging over vast distances, wandering albatross chicks almost always return to their colony of origin to breed^24^. Despite this high philopatry, wandering albatross populations separated by 1000 s of kilometres appear to be genetically homogenous^25^. This implies that they could have overlapping at-sea distributions, because in other seabirds, genetic divergence often reflects spatial segregation during the nonbreeding period^26^,^27^.

Here we used tracking data from an extensive sample of individuals to examine variation in migratory behavior and distribution during the sabbatical year within and between two genetically-similar populations of wandering albatross that breed 1000 km apart in the western Indian Ocean. Results are discussed in terms of the consequences for fitness.

## Results

**Figure 1:**
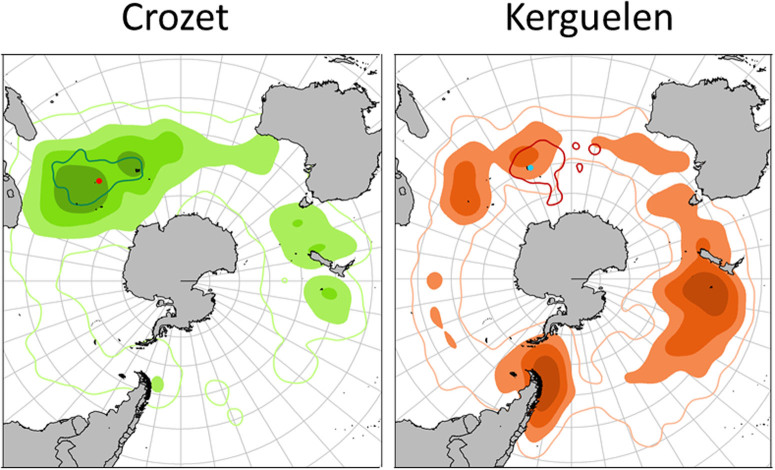
Kernel densities or utilisation distributions (25%, 50% 75% and 95% UDs) of adult wandering albatrosses tracked during the sabbatical year from Crozet (red dot) and Kerguelen (blue dot). The 95% UD of breeding birds is indicated by the solid line for each population (Figures produced from R software).

During the breeding season, wandering albatrosses tracked from Crozet and Kerguelen foraged in waters around the breeding grounds to maximum ranges of c. 3500 km (Fig. 1). When the breeding season ended in November, birds from the two populations dispersed throughout the southern oceans, but core areas were very different between populations (Fig. 1). Overlap between the two populations was low not only in the breeding season, but also during the sabbatical year (Fig. 1). However, within each population, the overlap in feeding zones used during the breeding and sabbatical year was near-complete for birds from Crozet, but minimal for those from Kerguelen birds (Fig. 1).

**Figure 2:**
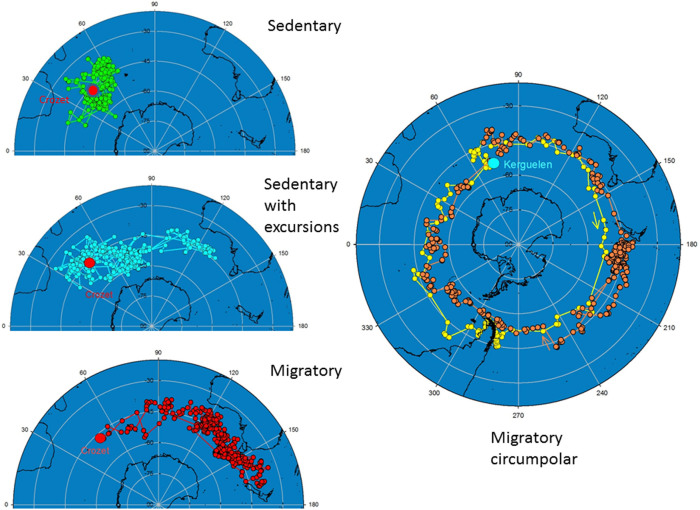
The three main strategies used by wandering albatrosses during sabbatical years, sedentary, sedentary with distant incursions of two Crozet birds, and migratory with two consecutive circumpolar movements of a Kerguelen bird: after an initial rapid flight to Chilean waters where the bird spent 2 months, it moved eastward through the Atlantic and the Indian Ocean (yellow track) to reach the Chatham Rise, east of New Zealand, before returning to Kerguelen (orange track) through the Pacific and Atlantic (Figure produced from Sigmaplot).

When considering individual movements, three distinct, site-specific strategies are found during the sabbatical year (Table 1)(χ^2^_2_ = 42.7, P<0.0001). Birds are either typically ***migratory***, with distinct wintering grounds at long distance from colonies, or ***sedentary***, spending either all the sabbatical year around the breeding grounds, or most of this time in this area but with one or two short term (<one month) but long-range excursions (***sedentary with excursion***) outside the western Indian Ocean (Fig. 2). All except one bird from Kerguelen were migratory, departing the Indian Ocean after breeding and, in almost all cases, circumnavigating Antarctica in an eastward movement with the westerlies to the Pacific Ocean (Fig. 2, Table 1), to winter off New Zealand and Chile (87%); the remainder wintered off Australia. Conversely, the majority of individuals from Crozet (72.9%) remained in the western Indian Ocean, mainly over deep waters, but also partly over the Crozet and Kerguelen shelves (Figs 1 and 2). They either were *sedentary* (40.2%) or *sedentary* *with excursions* to Australian waters or the central Atlantic Ocean (32.7%); the remaining 27.1% of Crozet birds were migratory. Of the latter, 44.8% migrated to southern Australia and the Tasman Sea where they spent the rest of the nonbreeding season before returning to Crozet, most returning against the prevailing westerly winds. The remainder circumnavigated Antarctica eastward with the westerlies, and, similar to the birds from Kerguelen, spent much of the non-breeding period in the Pacific off eastern New Zealand or Chile (Table 1, Figs. 1–2). These migrants from Crozet are mainly males (χ^2^_2_ = 10.2, P<0.001) (Table 1). There was no effect of year or age on the incidence of migratory or sedentary behaviours. All twelve birds tracked during two different sabbatical years separated by at least one breeding season, were consistent in their individual strategy, and those that were *migratory* (4) or *sedentary with excursions* (4) used the same foraging zones on each occasion.

Birds wintering east of New Zealand returned to Crozet or Kerguelen through the Pacific, covering on average 21,000 km. This was a detour of 13,000 km to avoid using the direct return route of 8,000 km against headwinds. It is noteworthy that 33% of birds from Crozet and Kerguelen that circumnavigated Antarctica repeated this journey twice during the same sabbatical year, and two birds completed three circumnavigations (Table 1, Fig. 2). Double and triple circumnavigations were performed by birds that moved first to Chile, where they stayed two months, and then, with tail winds, to New Zealand through the Drake Passage and the Atlantic and Indian oceans, covering a minimum of 22,000 km, i.e., a detour of >15,000 km instead of directly flying 7,000 km againts headwinds through the Pacific (Fig. 2). As a result of these successive detours to take advantage of westerly winds, distance covered during the long-distance migrations of these birds that made double or triple circumnavigations ranged between 79,000 and > 120,000 km, by far the longest migratory route of any animal studied to date. A single circumnavigation involved travelling 40,000 km on average, and the distances covered during shorter migrations ranged from 12,000 km, to 60,000 km for a bird that moved to Chile but returned against headwinds to Kerguelen.

**Figure 3:**
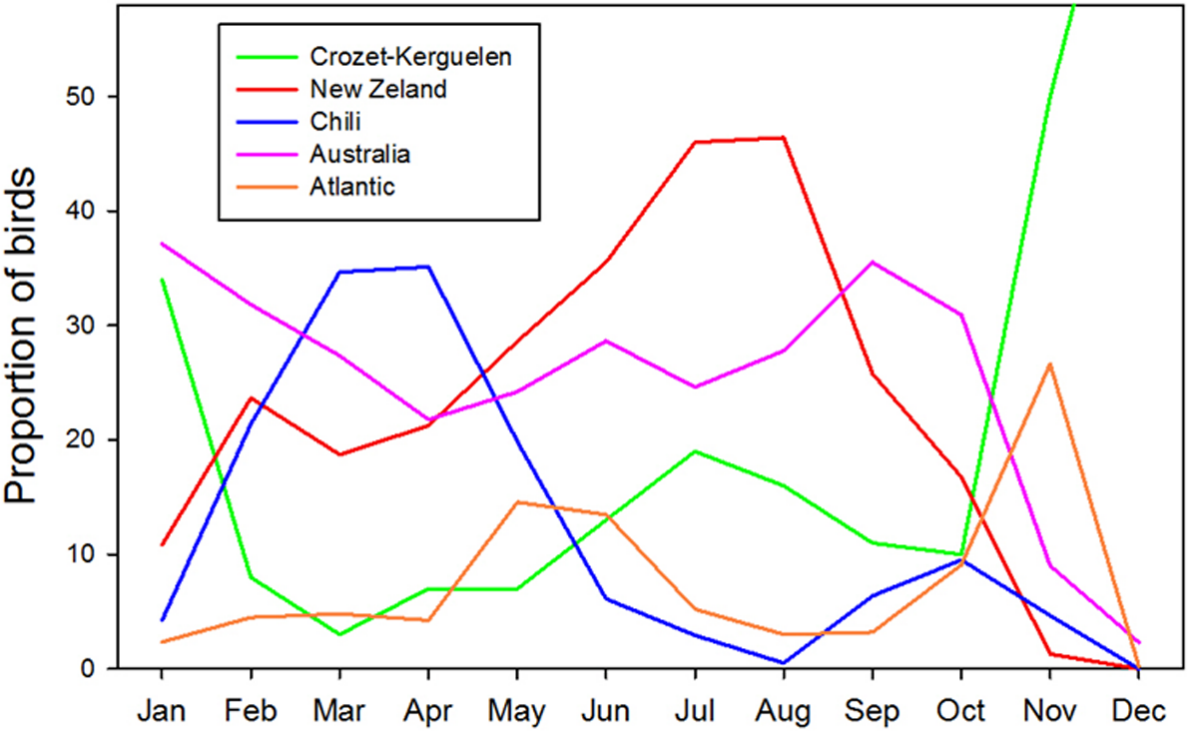
Proportion of migratory birds from Crozet and Kerguelen in the different oceanic sectors of the Southern Ocean over the annual cycle of the sabbatical year (Figure produced from Statistica).

Migrants left the Indian Ocean between mid-December and mid-January after fledging a chick, and returned in early December, having spent the sabbatical year off Australia or in the Pacific. Different ocean sectors were used seasonally by the circumpolar migrants; Chilean waters in March-April and New Zealand waters in July-September (Fig. 3). Wandering albatrosses spend the sabbatical year mainly in oceanic sub-tropical waters (Fig. 1), but on average females use waters that are 3°C warmer than males because they have a more northerly distribution (ANOVA F_1,131_ = 3.6, P = 0.031). Only for males did the mean temperature differ between sedentary (11.1°C) and migratory individuals (13.3°C). During the sabbatical year, sedentary and migratory birds differ in several aspects of their at-sea activity (flight) patterns. In South American waters (off Chile especially) where most migratory birds remained for an initial two months (Fig. 3), birds were very active, and spent substantially less time resting on the water than those off Australian and New Zealand (Table 2).

For birds tracked during the period 2000–2013, breeding success was similar for sedentary (two types) or migratory birds (F_2,116_ = 0.4, P = 0.664). However the proportion of birds that reared a chick and bred in the next calendar year instead of taking a sabbatical at least once between 2000 and 2013 was much higher (χ^2^_2_ = 13.1, P = 0.009) for those that were sedentary (20.9%) than for the migrants (4.2%). Similarly, at Kerguelen, where almost all birds are migratory and leave the southern Indian Ocean after rearing a chick, virtually the entire population shows a biennial breeding regime (99.6%, n = 335). In contrast, at Crozet, where birds are mainly sedentary and do not make excursions, a significant proportion of the population breed again within 1–2 months of fledging a chick (4.6% of birds are annual breeders, n = 2236, χ^2^_1_ = 12.9, P = 0.003). The majority (66.1%) of these annual breeders are females (Fisher Exact test P = 0.013). When taking a sabbatical year after rearing a chick, the proportion of birds changing partner was only 4.1%, whereas of those that breed again immediately, 20.1% of birds changed partners (χ^2^_1_ = 113.5, P<0.0001), particularly females rather than males (χ^2^_1_ = 8.1, P = 0.0045), and biennial breeders (χ^2^_1_ = 17.7, P<0.0001). Most (88.4%) changes of partner by annual breeder are temporary, and the bird returns to its original partner in the following breeding season, whereas in birds taking a sabbatical year, changes in partners are mostly (67%) definitive (χ^2^_1_ = 34.5, P<0.0001), due to the death of the original partner.

Finally, 24 sets of parent and offspring were tracked from Crozet during a sabbatical year. Of the seven parents that were migratory, 3 of their offspring were also migrants, and of the 17 sedentary parents, 13 of the offspring were sedentary, suggesting that strategies of offspring were not related to that of parents.

## Discussion

Our results show an astonishingly wide range of movement strategies during the sabbatical year in wandering albatrosses, from purely sedentary individuals that restrict foraging year-round to waters around the breeding colony, to others that have amongst the longest migratory routes known in animals. These differences exist between two populations of wandering albatrosses breeding only 1000 km apart that are known to be genetically very similar^25^. Even more surprisingly, this extreme variation occurs within the same population at the Crozet Islands. This represents partial migration, a pattern that is widespread in several animal taxa^5^ and was found recently in another seabird, where some males were sedentary whereas the rest of the population was migratory^22^. In wandering albatrosses, adoption of one of these alternative strategies - either migratory or sedentary - probably is not a heritable character, as related individuals often did not share the same behaviour. Instead, the individual strategy probably develops from experience during the long immature stage when birds have to learn foraging skills and locate productive waters to which they return subsequently. Once recruited into the breeding population, the behaviour (migratory or sedentary) and non-breeding zones visited by individual migrants during the sabbatical period remain the same over multiple years, and probably for life, supported by evidence from banding studies of multiple recaptures of the same birds in the same wintering zone^19^,^21^. This suggests that in wandering albatross, partial migration is not facultative at the individual level, in comparison with other species where the same individual may migrate in some years, but not in others^28^. Finally, strong sex-specific differences existed in the tracked population. First, as in the breeding season^29^, females foraged in warmer waters during the sabbatical year than males^30^. Second, males were much more likely to be migratory than females in the Crozet population, with important consequences in terms of fitness and mating behaviour. Similarly, sex-specific differences exist from the early stages after fledging, with longer migratory-type movements much more common in male than female juveniles from Crozet^31^,^32^.

The extreme variation in migratory strategies has profound effects in terms of resource and habitat use, as well as in energy budgets and, ultimately, important consequences for fitness, a crucial aspect that is generally difficult to assess without long term monitoring of individual breeding histories^33^. Our results show that the occurrence of migratory and sedentary behaviours during the non-breeding season of the two populations, and within the Crozet population, has several important implications in evolutionary terms. Although breeding success on average was similar in migratory and sedentary birds, the breeding frequency differed. Biennial breeding appears to be the strict rule in the Kerguelen population which is entirely migratory, and in migratory individuals from Crozet. Conversely, in sedentary birds that remain close to the breeding grounds year-round, a significant part of the population is able to breed annually^18^. Fitness costs of long-distance movements may be important for migratory animals^6^, some wandering albatrosses in this study covered even longer migratory distances than the longest recorded so far^11^,^15^ The migrants were mainly males, which might incur higher lifetime fitness costs than females: this sex-specific difference could explain the higher rate of senescence in males than females observed in the Crozet population for which there is, as yet, no clear explanation^34^. In addition, albatrosses are usually considered to be faithful to their partner through their life^35^, particularly in biennial species such as the wandering albatross^36^. However we found that to be able to breed annually, birds, especially females, change partner temporarily. Females are known to invest less in chick rearing, and may stop provisioning the chick several months before the male parent^37^. These females are thus able to start a new breeding attempt the following year if they are sedentary. Conversely, males provision the chick until it fledges, and would have much less time to recover. Indeed the condition of males deteriorates during the late phase of the chick provisioning period^38^ and bird need to reach a sufficient level of body condition before they can breed again^39^. As recovery of condition in one month would seem to be extremely unlikely, at Kerguelen, where almost all birds are migratory, annual breeding is exceptional.

As far as we are aware, the interaction between environmental conditions in local and distant habitats, and of individual attributes in shaping the co-existence of contrasting migratory tactics within a population remains poorly understood, and has been studied recently only in ungulates^5^,^28^. In wandering albatrosses, the habitats and thus marine resources used by sedentary birds in the western Indian Ocean are very different from those in the Pacific Ocean or off Australia in terms of water masses and other aspects of oceanography. Probably as a consequence, foraging strategies (activity patterns) in different habitats appear to vary; sedentary wandering albatrosses spent more time in flight and have higher energy expenditure (based on the rate of take-offs^40^), i.e., have a greater search effort than those migrating to Australian or New Zealand waters (Table 2). These latter sectors, where activity seems to be lower, might correspond to moulting zones. Indeed wandering albatrosses moult part of their flight feathers during the sabbatical year^41^, and could reduce activity at this time because of the increased flight costs associated with higher wing loading (smaller wing area). Conversely, in Chilean waters that are used only in March-April, the birds probably take advantage of a seasonally abundant resource that requires higher activity budget and costs, before moving to Australia and New Zealand waters. Alternatively, variable activity patterns might correspond to different foraging techniques related to different prey types. Another important implication of the differences in migration strategy is that by remaining during the sabbatical year in waters around the colony, sedentary birds from Crozet completely overlap with breeding birds (Fig. 1), whereas migratory birds from Kerguelen or Crozet do not overlap with breeders, and are only present around the colonies during the last month of the sabbatical year shortly before the new breeding season (Fig. 1).

Irrespective of the colony of origin, migration routes were mainly eastwards, i.e., following the westerly winds. It is important to note that birds which migrate only as far as Australia all return westward against the westerlies, whereas those that travel to the Pacific, continue to move eastward with the westerlies, making one or more circumnavigations of Antarctica. Thus New Zealand appears to be an important limit between Australia (Tasman Sea) and western Pacific (East of New Zealand). Longer circumnavigation (of 20,000 km) appears to be favoured by wandering albatrosses over the much shorter (< 8000 km) return trips against prevailing winds. A 12,000 km detour is probably an extreme case for a migratory bird^7^ but is not completely surprising since flying against the wind is much more costly than flying with the wind for albatrosses^13^,^42^. There is an interesting parallel with nonbreeding grey-headed albatrosses *Thalassarche chrysostoma* from South Georgia; these show highly variable migration strategies but any bird that moves east of Kerguelen (cf. the limit in the wandering albatross) never returns westward but instead continues on a circumpolar trajectory^15^.

The results of this study have important implications in terms of conservation of this threatened species. First, wandering albatrosses, as many other albatrosses and petrels, are threatened by longline fisheries^43^,^44^. Since each population has a very different distribution during the sabbatical years, they are exposed to different fisheries; those operating over the continental shelves of South America and New Zealand for Kerguelen birds, and over deep oceanic waters or the Crozet and Kerguelen oceanic shelves for Crozet birds, representing different threats in terms of the fishing methods, spatio-temporal variation in effort, and levels of bird bycatch mitigation used by different fleets, and also in terms of the relative availability of discards which are used by this scavenging species. Similarly, even within the Crozet population, individuals with different strategies are susceptible to varying levels of fisheries overlap, potentially with implications for the dynamics of the population^45^.

Second, we know that climate change is already affecting the Crozet population due to changes in wind conditions during breeding^29^. Since migration routes of wandering albatrosses are located within a narrow latitudinal range (35 to 55°S), corresponding to the present main flow of the westerlies wind (Fig. 1), it is likely that the ongoing changes in wind condition in the Southern Ocean, especially the southward shift of the westerlies^46^ have the potential to affect migratory strategies. The evolution of seasonal migration in birds has facilitated diversification through the divergence of migratory subpopulations^47^. Thus it is possible that the pattern of partial migration observed today corresponds to a unusual situation for an oceanic seabird, in which the population is responding to climatic change, potentially with one set of tactics being progressively replaced by another^48^. At Crozet, this could explain the dominance of a sedentary non-breeding strategy in recent decades. Historical data from Australia where Crozet birds were known to spend their sabbatical year in large number during the 1960–1970s^21^ show that numbers have progressively declined to very low levels due to a reduction in availability of a local food resource^49^. Thus the migratory type may be replaced progressively by birds with a sedentary strategy; however, this would need to be confirmed by future monitoring of the proportions of sedentary and migratory birds in the population that are selected during the juvenile phase. Thus this study illustrates how a better understanding of the evolution and coexistence of different life-history strategies within populations can help us to better understand the evolution of migration^50^.

## Methods

The methods were carried out in accordance with the approved guidelines of the Reserve Nationale des Terres Australes for the capture of live protected animals. The procedures used to capture and handle birds, and attach loggers were approved by The Préfet des Terres Australes and Antarctiques Françaises, and by the Ethic Committee of the French Polar Institute (IPEV). The study was carried on wandering albatrosses at the Crozet (46°S, 52°E) and Kerguelen Islands (50°S, 70°E) where long term monitoring, based on annual mark-recapture studies have been carried out annually since 1966 and 1995, respectively^44^. Most birds were of known age and breeding experience. The incidence of annual and biennial breeding after a successful breeding attempt, and fidelity to the partner, were estimated between the period 2000 and 2012 for 2236 individuals at Crozet and 335 individuals at Kerguelen. We tracked wandering albatrosses from Crozet and Kerguelen during the sabbatical year using Global Location Sensing (GLS) - immersion loggers (British Antarctic Survey, Cambridge; 1998–2013. The loggers (2.5 g) were fixed with cable-ties to a plastic leg band and retrieved after one or several years. A total of 180 individuals were fitted with GLS between 2007 and 2013 and, so far, 134 loggers were recovered one to 4 years later, and the data downloaded (Table 1). In addition, between 1989 and 2012, a total of more than 400 albatrosses of known sex and age were equipped with either Argos Platform Terminal Transmitters (PTTs) or GPS loggers (2002–2013) to record movements during the breeding season^29^. Birds were captured before taking off for the sea, loggers attached with adhesive tape to the back feathers, and the devices retrieved after one or several foraging trips. Details of equipment and analysis of PTT and GPS data are given in^13^,^31^. The total mass of devices was always far below the recommended 3% threshold^51^. Long term analysis of the potential effects of instrument deployment shows no negative impacts on breeding success and frequency, or survival^52^.

GLS light data were analyzed following^11^,^53^,^54^ to estimate locational information accurate to 170 km on average^54^. In addition to light levels, GLS loggers recorded salt water immersion, allowing the estimation of activity from periods spent in flight or sitting on the water, as well as the number of changes in state (take-offs, and landings, that are known to be the main driver of energy expenditure^40^). The loggers also recorded in situ sea surface temperature that were used to correct latitude estimates^55^. As positions obtained from geolocators have a relatively low accuracy, 1) only distance covered during migratory routes when birds showed a rapid directional longitudinal movement (see 12 were estimated, 2) we used a conservative approach to filter data according to maximum speeds and calculate distances covered excluding days with mean speeds > 40 km/h, since birds spend on average > 50% of time daily sitting on water (see results). Therefore values presented for total distance covered during migration should be regarded as minimum estimates. Movements during the sabbatical year were considered as migratory in the case of a persistent directional movement from the breeding grounds and its surrounding waters to one (or several) distant zone(s) where the individual spend more than one month in each zone.

Statistical analyses were performed in STATISTICA 12.0. Tests were two tailed, and the presented values mean ± S.D. We estimated location density distribution maps using fixed kernel density using the ad hoc method of the ‘adehabitat' package (i.e. bivariate normal kernel) using software R.

## Author Contributions

H.W. designed the study, organized field work, performed the research and wrote a first version of the manuscript. K.D., A.G. and P.P. managed data and performed analyses. H.W., K.D. and R.P. contributed substantially to revisions of the manuscript, and R.P. corrected the English.

## Figures and Tables

**Table 1:** Migration strategies of wandering albatrosses tracked from Crozet and Kerguelen during the sabbatical year

Population	N tracked	Sedentary in western Indian Ocean	Sedentary with incursion(s) to Australia or Atlantic	Migratory	Numbers of circumpolar tours of migratory birds (%)			
					0	1	2	3
Crozet	107	40.2%	32.7%	27.1%	55.2	20.7	20.7	3.4
Kerguelen	24	0	4.2%	95.8%	4.4	47.8	39.2	4.4

**Table 2:** Activity patterns on the wintering grounds (excluding the migratory movements) of wandering albatrosses tracked from Crozet and Kerguelen during the sabbatical year

Behaviour on wintering grounds	Sedentary (151)	Migratory Australia New-Zealand (141)	Migratory South America (33)	test
Activity (% time on water)	65.5 ± 9.7	68.2 ± 10.3	66.4 ± 12.5	F_2,116_ = 2.2 P = 0.108
Activity at night % time on water	72.5 ± 11.0	77.1 ± 11.6	72.4 ± 20.5	F_2,116_ = 3.6, P = 0.030
Mean proportion time resting at night (%)	60 ± 11.9	65.6 ± 14.4	61.7 ± 20.8	F_2,116_ = 3.8, P = 0.024
Number of daily take-offs during day time	14.4 ± 4.1	12.2 ± 4.2	17.2 ± 6.1	F_2,116_ = 14.7, P < 0.0001
